# Health of Philadelphia

**Published:** 1845-05

**Authors:** 


### THE MEDICAL EXAMINER.

### PHILADELPHIA, MAY, 1845.

### HEALTH OF PHILADELPHIA.

We have never known the measles so prevalent in Philadelphia as at the present time. From the reports of the Board of Health, and all that we can learn of the experience of our friends, as well as from our own observation, the epidemic appears to be characterized by great mildness—we have certainly never seen it more so. We have also some cases of erysipelas. This disease, although not at all general, like rubeola, in the instances that we have seen, has been marked by unusual violence. The constitutional affection, of which the eruption must be regarded as merely a manifestation, is of a decidedly adynamic character. Generally there has been no affection of the throat; but in two instances that have come under our notice, the disease commenced with a swelling of the tonsils and soft palate, and also of the tongue, to which the efflorescence on the face and head quickly succeeded. Of latter years, a connexion between erysipelas and epidemic puerperal peritonitis has been supposed to exist, by many excellent observers, but the question is by no means settled. We have recently had a good many cases of the latter disease in various parts of the city; and although they have seemed to be sporadic, they have been exceedingly severe, and far more fatal than is usual for accidental cases, or such as occur without the presence of an epidemic cause. One of the most rapidly fatal of the cases that have come to our knowledge, occurred in the practice of a gentleman who, at the time when he delivered the patient, was in almost contant attendance upon a patient with malignant erysipelas. We mention the circumstance to direct to it the attention of our brethren who are engaged in the practice of midwifery, that they may notice the facts that bear upon this deeply interesting point.

An able article on the nature, origin, and treatment of puerperal fever, by Dr. Blackmore, of Edinburgh, will be found among the articles of our *Record* in the present number. Whatever may be thought of the conclusions of Dr. B. touching the malignant and specific character of *sporadic* cases of puerperal fever, our experience teaches us the necessity of perfect non-intercourse between the sick and those who are liable to the disease, and particularly in Hospitals and Lying-in Institutions.

